# Transcutaneous Electrical Nerve Stimulation on the PC-5 and PC-6 Points Alleviated Hypotension after Epidural Anaesthesia, Depending on the Stimulus Frequency

**DOI:** 10.1155/2012/727121

**Published:** 2012-01-31

**Authors:** Young-Chang P. Arai, Akihiro Ito, Kenji Ohshima, Soki Hibino, Sinnosuke Niwa, Jun Kawanishi, Hiroki Numanami, Yoshikazu Sakakima, Shouji Mizuno, Yusuke Tawada, Yuki Maruyama, Jun Sato, Makoto Nishihara, Shinsuke Inoue, Takahiro Ushida

**Affiliations:** ^1^Department of Surgery, Toki Municipal General Hospital, Gifu 509-5193, Japan; ^2^Multidisciplinary Pain Centre, Aichi Medical University, School of Medicine, 21 Karimata, Nagakutecho, Aichigun, Aichi 480-1195, Japan

## Abstract

Neuraxial blockade causes arterial hypotension. Transcutaneous electrical nerve stimulation (TENS) at the Neiguan (PC-6) and Jianshi (PC-5) reduces the severity of hypotension after spinal anaesthesia, but did not clarify the optimal stimulus frequency. We hypothesized that the stimulus frequency of TENS at the PC-6 and PC-5 points would influence the severity of hypotension after epidural anaesthesia. 65 ASA I or II male patients presenting for inguinal hernia repair were randomized to five groups: the control group received no treatment; the 2 Hz, 10 Hz, 20 Hz, and 40 Hz groups received TENS at a frequency of 2 Hz, 10 Hz, 20 Hz, and 40 Hz, respectively. The lowest SBP was significantly higher in the 40 Hz group [the control, 84 (74–110) mmHg; the 2 Hz, 96 (62–116) mmHg; the 10 Hz, 100 (68–110) mmHg; the 20 Hz, 96 (64–115) mmHg; the 40 Hz, 104 (75–140) mmHg: *P* = 0.004]. Significantly less patients experienced hypotension in the 40 Hz group [the control, 78%; the 2 Hz, 43%; the 10 Hz, 38%; the 20 Hz, 38%; the 40 Hz, 8%: *P* = 0.008]. TENS on the PC-6 and PC-5 points reduced the severity and incidence of hypotension after epidural anaesthesia, depending on the stimulus frequency.

## 1. Introduction

Neuraxial blockade, epidural anaesthesia, causes arterial and venous vasodilation and decrease in venous return due to blockade of the sympathetic nervous system, which results in arterial hypotension [1, 2].

 In animal studies, electroacupuncture at the Neiguan (PC-6) and Jianshi (PC-5) points affects the circulatory and sympathetic nervous system [3, 4], especially electroacupuncture at the PC-6 point increases haemodynamics [5] and makes bleeding-induced hypotension less severe [6]. Our previous study showed that a frequency of 50 Hz of transcutaneous electrical nerve stimulation (TENS) at the PC-5 and PC-6 reduces the severity of hypotension after spinal anaesthesia, but did not test the appropriate frequency of electrical stimulation of TENS [7]. However, several studies showed that electroacupuncture evokes pressor or depressor response, depending on the stimulus frequency. That is, the autonomic nervous system might be influenced by the stimulus frequency of TENS.

 We thus hypothesized that the stimulus frequency of TENS at the PC-6 and PC-5 points would affect the activity of the autonomic nervous system, thereby differentially influencing the severity of hypotension after epidural anaesthesia. The purpose of the present study was to test the effect of four different frequencies of TENS at the PC-5 and PC-6 points on haemodynamics after epidural anaesthesia in patients undergoing inguinal hernia repair.

## 2. Methods

After obtaining approval from the Ethics Committees of our institutions and written informed patient's consent, 67 ASA I or II male patients presenting for inguinal hernia repair under epidural anaesthesia were enrolled in the present study. Patients suffering from hypertension, diabetes, or obesity were excluded.

**Figure 1:**
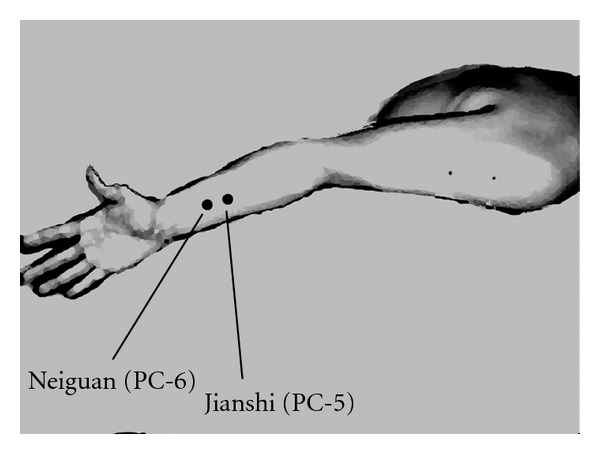
The locations of small-sized cutaneous electrode pads for transcutaneous electrical nerve stimulation.

Patients were randomized into five groups, using sealed envelopes: the control group received no treatment; the 2 Hz group received TENS at a frequency of 2 Hz bilaterally at the PC-5 and PC-6 points (on the palmar side of both arms, between the tendon of the long palmar muscle and radial flexor muscle of the wrist) [7, 8] (Figure 1) by a TENS stimulator (NeuroTrax TENS & AcuStim; Verity Medical LTD, Hampshire, uk); the 10 Hz, 20 Hz, and 40 Hz groups received TENS at 10, 20, and 40 Hz bilaterally at the PC-5 and PC-6 points by the TENS stimulator, respectively. All patients fasted for a minimum of 6 h preoperatively. At the operation room, all patients had standard monitoring in place (noninvasive arterial pressure, electrocardiogram (ECG), and pulse oximetry) and these baseline values were recorded. In the 2 Hz, 10 Hz, 20 Hz, and 40 Hz groups, then, small-sized (1.5 cm) cutaneous electrode pads were put bilaterally at the PC-6 and PC-5 points. The intensity of the electrical stimulation was adjusted to produce the most intense tolerable electrical sensation without muscle contractions at a frequency of 2, 10, 20, or 40 Hz and a duration of 100 *μ*s until the end of surgery. After intravenous access, acetated Ringer's solution (10 mL kg^−1^) was administered before the induction of anaesthesia. With the patient on the right side, 10 mL of 2% lidocaine was injected after identification of the epidural space at the L1-L2 interspace using a 17-gauge Tuohy needle, and then a 20-gauge epidural catheter was placed.

 The anaesthetic level was measured by pinprick at the right mid-clavicular line every two minutes. Surgery started when an adequate dermatome level of anaesthesia from Th8 to L2 was ensured. In the case of inappropriate cephalad spread of anaesthesia, incremental epidural supplements of 2% lidocaine were given, starting with 4 mL. When necessary, additional 2 mL boluses were given no earlier than 5 min after the preceding top-up. Haemodynamics were recorded every minute for 30 minutes after the injection of 10 mL of 2% lidocaine and then every 2.5 minutes until the end of surgery. In the case of hypotension, ephedrine of 4 mg was given intravenously. If necessary, additional ephedrine of 4 mg was given every two minutes. We defined hypotension as a decrease in systolic blood pressure (SBP) 30% below baseline values or to less than 90 mmHg in the present study. Atropine of 0.5 mg was given intravenously to treat bradycardia, defined as a decrease in heart rate to less than 50 beats min^−1^.

Using empirical data from our daily clinical practice, the mean (SD) of the lowest SBP was 85 [9] after epidural injection of 10 mL of 2% lidocaine. Thus, a group size of at least 12 patients was needed to show a difference of 15 (SD 10) in the lowest SBP with a significant level of 0.05 (*α* = 0.05) and a power of 80% (*β* = 0.20). Data are expressed as median (range). Statistical analysis was performed using the Kruskal-Wallis test followed by Dunn's method for multiple comparisons. A *P* < 0.05 value was considered to be significant.

## 3. Results

Demographic data and baseline measurements to wound closure time were comparable among the five groups (Table 1). Baseline systolic blood pressures (SBPs) were similar among these groups (Table 2).

 The lowest SBP was significantly higher in the 40 Hz group compared to that of the control, 2 Hz and 20 Hz groups (the control, 84 (74–110) mmHg; the 2 Hz, 96 (62–116) mmHg; the 10 Hz, 100 (68–110) mmHg; the 20 Hz, 96 (64–115) mmHg; the 40 Hz, 104 (75–140) mmHg, *P* = 0.004) (Table 2). Significantly less patients experienced hypotension in the 40 Hz group (the control, 11 (78%); the 2 Hz, 6 (43%); the 10 Hz, 5 (38%); the 20 Hz, 5 (38%); the 40 Hz, 1 (8%), *P* = 0.008). Two, one, and four patients experienced severe hypotension (SBP less than 70 mmHg) in the 2 Hz, 10 Hz, and 20 Hz groups, respectively. Less ephedrine was required to maintain arterial blood pressure in the 40 Hz group (the control, 8 (0–16) mg; the 2 Hz, 0 (0–20) mg; the 10 Hz, 0 (0–8) mg; the 20 Hz, 0 (0–12) mg; the 40 Hz, 0 (0–8) mg, *P* = 0.013).

## 4. Discussion

The present study showed that a frequency of 40 Hz of TENS on the PC-5 and PC-6 points significantly reduced the severity and incidence of hypotension after epidural anaesthesia in patients undergoing inguinal hernia repair, compared to frequencies of 2, 10, and 20 Hz of TENS on the two points.

 Acupuncture, acupressure, and TENS on the traditional acupuncture points have been administered for peri-operative management [9–11]. Electroacupuncture at an acupuncture point affects haemodynamics and the sympathetic nervous system [8, 12, 13]. Electroacupuncture at the PC-5 increases stroke volume and cardiac output and furthermore reduces the severity of bleeding-induced hypotension [5, 6].

 Since TENS was administered at the most intense tolerable electrical sensation in the present study, there is a possibility that TENS itself had some activating effects on the sympathetic nervous system [7]. While administering the same level of electrical stimulation, however, TENS at the specific frequency, 40 Hz, significantly sustained blood pressure. Some studies showed that electroacupuncture on the PC-5 at 40 Hz enhances myocardial function and prevents bleeding-induced hypotension [5, 6], which are consistent with the present study. In contrast, electoacupuncture at 2–4 Hz suppresses cardiovascular sympathetic reactions [3, 4]. In fact, 2, 10, and 20 Hz stimuli induced severe hypotension in some patients in the present study. A stimulation of somatic afferent fibres at 5 Hz causes a depressor effect, while a stimulation of 40 Hz leads to a pressor effect [14]. Also, a study showed that stimulation of myelinated fibres alone or myelinated and unmyelinated fibres together leads to a depressor effect [15]. In contrast, stimulation of unmyelinated fibres alone causes a pressor effect. We thus speculate that 40 Hz stimulus might have provoked the activation of unmyelinated fibres, compared with 2, 10, and 20 Hz stimuli in the present study.

In conclusion, TENS on the PC-5 and PC-6 points reduced the severity and incidence of hypotension after epidural anaesthesia in patients undergoing inguinal hernia repair, depending on the stimulus frequency.

## Figures and Tables

**Table 1:** Demographic and anaesthetic data. Data are median (range). Wound-based time: baseline measurements to wound closure time.

	Control (*n* = 14)	2 Hz (*n* = 13)	10 Hz (*n* = 12)	20 Hz (*n* = 13)	40 Hz (*n* = 13)	*P*
Age (yr)	69 (61–74)	66 (43–82)	74.5 (49–88)	73 (23–84)	69 (36–886)	0.442
Height (cm)	160 (148–173)	163 (148–175)	159 (148–174)	162 (150–170)	159 (137–179)	0.773
Weight (kg)	61 (50–72)	62 (39–76)	57 (41–64)	58 (45–66)	54 (47–94)	0.578
Wound-based time (min)	45 (28–55)	40 (28–55)	42 (38–46)	42 (38–45)	40 (35–55)	0.932

**Table 2:** Haemodynamic data and dose of ephedrine. Data are median (range) or number (percentage). SBP: systolic blood pressure. ^†^: significantly different from the control group. *: significantly different from the 40 Hz group.

	Control (*n* = 14)	2 Hz (*n* = 14)	10 Hz (*n* = 13)	20 Hz (*n* = 13)	40 Hz (*n* = 13)	*P*
Intensity of TENS (mA)	—	13 (9–17)	12 (9–15)	13 (9–15)	13 (9–15)	0.721
Baseline SBP (mmHg)	130 (104–150)	135 (115–145)	130 (110–145)	130 (114–145)	132 (116–158)	0.994
Lowest SBP (mmHg)	84 (74–110)*	96 (62–116)*	100 (68–110)	96 (64–115)*	104 (75–140)	0.004
Baseline HR (beats min^−1^)	72 (56–98)	76 (56–105)	70 (60–85)	70 (54–85)	75 (55–105)	0.343
HR at lowest SBP (beats min^−1^)	62 (45–80)	64 (40–76)	61 (44–86)	55 (45–75)	65 (50–95)	0.522
Incidence of hypotension (*n*)	11 (78%)	6 (43%)	5 (38%)	5 (38%)	1 (8%)^†^	0.008
Ephedrine (mg)	0 (0–16)	8 (0–20)	0 (0–8)	0 (0–12)	0 (0–8)^†^	0.013
